# Depressive Symptoms of Centenarians during the COVID-19 Pandemic: Preliminary Results of an Exploratory Study in Switzerland

**DOI:** 10.1192/j.eurpsy.2022.1663

**Published:** 2022-09-01

**Authors:** C. Gomes Da Rocha, A. Von Gunten, K. Uittenhove, C. Lampraki, S. Cavalli, F. Herrmann, D. Jopp

**Affiliations:** 1 HES-SO Valais-Wallis , School Of Health Sciences, Sion, Switzerland; 2 University of Porto, Institute Of Biomedical Sciences Abel Salazar, Porto, Portugal; 3 Lausanne University Hospital, Service Of Old Age Psychiatry, Prilly, Switzerland; 4 University of Lausanne, Institute Of Psychology, Lausanne, Switzerland; 5 University of Lausanne, Swiss National Centre Of Competence In Research Lives, Lausanne, Switzerland; 6 University of Applied Sciences and Arts of Southern Switzerland (SUPSI), Department Of Business Economics, Health And Social Care - Centre Of Competence On Ageing, Manno, Switzerland; 7 Geneva University Hospitals - Trois-Chêne Hospital, Department Of Rehabilitation And Geriatrics, Thônex/Geneva, Switzerland

**Keywords:** depressive symptoms, Covid-19, centenarians

## Abstract

**Introduction:**

Depression is one of the most frequent mental health problems in older populations.^1^ To the best of our knowledge, the prevalence of depressive symptomatology (DS) among centenarians in Switzerland is unknown. Furthermore, the COVID-19 pandemic may have had a negative impact. As part of the study SWISS100^2^, we intend to provide key information on centenarians’ levels of DS.

**Objectives:**

To describe the DS of Switzerland’s centenarians during the COVID-19 pandemic.

**Methods:**

Randomly selected centenarians from across Switzerland and their proxy relatives were invited to participate. Data are collected via telephone. The questionnaire includes the assessment of DS via the Geriatric Depression Scale (GDS)– 5 items.^3^ Preliminary data were analysed using descriptive statistics.

**Results:**

Telephone interviews were completed with 51 centenarians, and for 19 of them, proxy relatives also answered. The M_Age_ of the centenarians was 101.41 (1.47) years, 34 (66.67%) were female and 27 (52.94%) lived at home. The mean score of the GDS–5 was 1.32 (SD=1.49). Considering a cut-off ≥2, 18 (36%) centenarians were screened positive for possible depression. Descriptive statistics indicated effects of gender (men: M=1.41, SD=1.46; women: M=1.27, SD=1.53) and living situation (private: M=1.07, SD=1.36; institution: M=1.61, SD=1.62). Centenarians’ and proxy reports were significantly related (ρ=0.56; p<.05).

**Conclusions:**

Clinically relevant DS are highly prevalent among centenarians during the COVID-19 pandemic (36%), which is consistent with a recent study^4^ reporting a prevalence of 32% in a sample of younger older adults (M_Age_: 77.6, SD=6.9). To conclude, DS in centenarians should be screened systematically, especially in this time of unprecedented health crisis.

**Disclosure:**

No significant relationships.

